# World postpartum haemorrhage day: Renewing the global call to end deaths from postpartum haemorrhage

**DOI:** 10.1002/pmf2.70130

**Published:** 2025-10-03

**Authors:** PPH Roadmap Advocacy Working Group

## 1 INTRODUCTION

Excessive bleeding after childbirth, known as postpartum hemorrhage (PPH), remains a leading cause of maternal mortality worldwide. Of the estimated 260,000 maternal deaths in 2023, nearly 45,000 were attributable to PPH [1]. In October 2023, the World Health Organization (WHO) and several partners launched the *Roadmap to Combat Postpartum Haemorrhage between 2023 and 2030*, a unifying strategy to galvanise action across PPH research, norms, implementation, and advocacy [2]. The Roadmap called on governments, funders, academia, professional societies, industry and innovators, women's groups and civil society to accelerate action for PPH priorities. While this call to action has mobilised the global community toward a more proactive PPH agenda, persistent implementation gaps, systemic inequities, and geopolitical stressors continue to limit progress. To spotlight PPH as a preventable cause of maternal death, a World PPH Day was proposed as an annual global advocacy activity to keep Roadmap objectives on track [3]. As we approach 5 October 2025, the inaugural World PPH Day, the maternal health community is uniting behind a renewed call to action. This commentary reflects on progress since the launch of the Roadmap, highlights lessons from country experiences, and outlines why urgent, coordinated action is necessary to realise its promise.

## 2 PROGRESS AND PERSISTENT GAPS

Global maternal mortality has declined by 41% between 2000 and 2023 [4]. Reductions in deaths from PPH have contributed significantly to this progress. Yet gains remain uneven. While high‐income countries have seen dramatic declines, women in sub‐Saharan Africa and South Asia continue to die at disproportionately high rates from PPH, despite availability of effective preventive, diagnostic and treatment measures. These deaths reflect systemic weaknesses, including overburdened health systems, fragile supply chains, and poor quality or inconsistent availability of essential medicines that leave providers unprepared for emergencies.

Proven solutions to PPH have failed to gain traction due to persistent gaps in policy and programme implementation that are compounded by inadequate domestic funding. Quality‐assured essential PPH medicines such as uterotonics, tranexamic acid (TXA), and iron supplements; trained providers; and functional emergency response systems, including blood transfusion facilities, are still not readily available in many high‐burden countries. Global stressors such as geopolitical instability, shrinking development funding, climate shocks, and persistent gender inequalities exacerbate these vulnerabilities. In fragile and conflict‐affected settings, women face the highest risks, as skilled birth attendance, life‐saving PPH commodities and emergency obstetric care are often inaccessible [4].

## 3 ACHIEVEMENTS OF THE PPH ROADMAP

Since its launch, the PPH Roadmap has catalysed progress across four strategic areas: research, norms and standards, implementation, and advocacy. One of the major achievements in research and evidence translation has been the publication and guideline uptake of EMOTIVE trial findings [5]. WHO promptly issued new recommendations on the PPH treatment bundle – largely informed by EMOTIVE trial findings – and on the use of objective blood loss assessment within six months of publication of the trial results [6]. Several countries including Nigeria, Ethiopia, Rwanda, India, Bangladesh, and Kenya have adopted the PPH treatment bundle into national policies, training programmes, and networks of care, marking an important step in translating evidence into practice.

Momentum is also building around other research priorities. Ongoing clinical trials are evaluating alternative routes of TXA administration and the use of heat‐stable carbetocin for PPH treatment, both of which could improve access to first‐response PPH treatment in resource‐limited settings [7]. Nearly all research priorities outlined in the innovation track of the Roadmap are now either underway or completed.

In line with the key objectives of the norms and standards strategic area, WHO, the International Federation of Gynecology and Obstetrics (FIGO), and the International Confederation of Midwives (ICM) have jointly developed a consolidated suite of evidence‐based recommendations and complementary tools to support country‐level implementation. This harmonized guidance and tools, to be formally launched on the first World PPH Day, provide a critical foundation for standardised care worldwide, and represents a unique model for collaboration among international guideline developers.

Country‐level implementation illustrates how system‐wide improvements can reduce PPH mortality. In Kenya, uterotonic stock monitoring has been integrated into supply chain systems. In Bangladesh, digital decision‐support tools for midwives now embed PPH algorithms to enable early recognition and standardised response. Pilot programs in Nigeria, Uganda, Ghana, Malawi, Nepal, Pakistan, Sierra Leonne, Ethiopia and India demonstrate how integrated approaches – linking reliable supply chains, midwife training, and community engagement – can significantly lower PPH‐related mortality.

Global advocacy has been strengthened through partnerships between professional societies, civil society, and UN agencies. The *PPH Roadmap Advocacy Working Group* has developed a global advocacy framework, laying the groundwork for World PPH Day. An existing learning and exchange platform, such as the PPH Community of Practice, has expanded to more than 70 organisations to further enhance collaboration and knowledge sharing across countries.

Despite these advances, several key areas of the Roadmap have not progressed as envisioned. Efforts to address implementation bottlenecks remain fragmented and largely donor‐driven. Without political leadership and sustainable domestic financing to scale PPH prevention, diagnosis and treatment, the momentum attained so far risks stalling.

## 4 CHALLENGES AND OPPORTUNITIES FOR SCALING

The PPH Roadmap has shown that rapid progress in evidence translation is possible, but challenges remain in scaling of solutions. Many countries are yet to institutionalise PPH treatment bundles or secure sustainable financing for medicines and training. Promising innovations such as heat‐stable carbetocin for PPH prevention and intravenous TXA for PPH treatment are underused. Many health systems still lack comprehensive strategies for prevention, diagnosis, and treatment of maternal anaemia, which places women at greater risk of death from PPH [8].

Implementation research, particularly on product access approaches, provider training, and health system integration, remains underfunded. Without clarity on generalizability of research findings beyond project sites, promising interventions risk remaining pilots rather than becoming national programmes.

The global funding environment has tightened in 2025, with donors scaling back their investments in maternal health. This creates urgency but also an opportunity to innovate, pool resources, and maximise efficiencies to achieve more with less. Strategic leadership from ministries of health and cross‐sector engagement will be vital in building resilient and sustainable ecosystems to end PPH mortality and morbidity.

Real‐time data and data science is critical for accountability. Tools such as maternal near‐miss audits, community scorecards, and digital dashboards can help detect PPH hotspots, direct resources, and support responsive policymaking. Strengthening of national health information systems must be a priority for all countries.

Emerging biomedical and service delivery innovations offer new hope for the future. Heat‐stable uterotonics, new uterine devices, tools for quantification of blood loss, blood transfer systems for rural settings, risk assessment tools, and tools to enhance midwifery models of care can dramatically improve outcomes. The PPH agenda could benefit from precision public health approaches that integrate data modeling, geospatial mapping, and predictive analytics for targeting individual‐ or population‐specific interventions.

## 5 WORLD PPH DAY: COMMEMORATION AND ADVOCACY

The establishment of World PPH Day is more than symbolic; it is a strategic act. It provides a platform to: honour the memory of the tens of thousands of women who die annually from PPH; mobilise governments, funders, and communities to commit resources; change policy; amplify women's and their families’ voices with survivor stories to humanise the issue; and drive awareness that PPH is a human rights and equity issue, not only a clinical emergency.

Each year, World PPH Day will spotlight progress and challenges, aligning advocacy with existing global commitments such as the Sustainable Development Goals (SDG) and Universal Health Coverage (UHC), and acceleration efforts such as *Every Woman, Every Newborn, Everywhere* (EWENE) initiative [9]. It will help break the pervasive silence around PPH as a preventable cause of maternal death, and chart a path forward that is inclusive, evidence‐based, and equity‐focused. At the country level, campaigns will include webinars, policy roundtables, community storytelling, survivor testimonials, and media engagements. Press kits, advocacy toolkits, and adaptable templates will be developed and distributed in advance to governments and partners to ensure consistent branding and messaging while enabling local adaptation. Additionally, the events will foster dialogues and discussions within the broader scope of maternal health on issues such as equitable access, gender norms, nutrition, climate change and social determinants of health.

October has been chosen strategically to align with the Roadmap's anniversary, creating an annual opportunity to review progress. Each year's theme will guide the day's focus and messaging, ensuring that advocacy efforts remain dynamic, relevant, exciting and strategic.

## 6 A RENEWED CALL TO ACTION

The maternal health community must seize the clarion call from the World PPH Day to transform the Roadmap's commitments into sustained action and ensure that PPH remains politically visible and globally relevant. In this inaugural year, we reiterate the Roadmap's call for all stakeholders to count, audit and account for every woman who dies from PPH, with the aim of driving corrective actions. Governments must set explicit PPH reduction targets, strengthen accountability and monitoring systems, update and disseminate national guidelines, ensure reliable supply of quality‐assured medicines and devices, and invest in staffing and training at all levels of care. Funders must expand and coordinate investments, aligning their portfolios to accelerate implementation and avoid fragmentation. Professional societies have a duty to champion the forthcoming WHO‐FIGO‐ICM consolidated PPH guidelines, lead continuous staff training and uphold standards of care.

Women and women's groups must be fully engaged as advocates and partners in shaping solutions, ensuring that action reflects lived realities. The research community must close the remaining priority knowledge gaps, especially in implementation and delivery, and translate findings swiftly into policy and practice. The tools are in our hands, the evidence is clear, and the solutions are known. What remains is our collective duty to act boldly and decisively, until no woman dies from PPH.

## CONFLICT OF INTEREST STATEMENT

Andrew Weeks received funding for a randomized trial of oxytocin versus carboprost for PPH treatment (ISRCTN16416766), and a patent and clinical trial of the PPH Butterfly—a new device to manage PPH (ISRCTN15452399). All other authors declare no competing interests.

## FUNDING INFORMATION

No funding was received by the authors for this editorial.

## PPH ROADMAP ADVOCACY WORKING GROUP

Members of the PPH Advocacy Working Group (listed in alphabetical order) and their affiliations are presented below:

**Table:** 

Name	Affiliations
Natalie A Bailey	UNDP/UNFPA/UNICEF/WHO/World Bank Special Programme of Research, Development and Research Training in Human Reproduction (HRP), Department of Sexual, Reproductive, Maternal, Child, Adolescent Health and Ageing, World Health Organization
Mailys Bobin	Unitaid
Manuel Couffignal	United Nations Population Fund (UNFPA)
Milka Dinev	Reproductive Health Supplies Coalition
Maria Donatelli	Unitaid
Daniella Drandic	International Confederation of Midwives
Jacqueline Dunkley‐Bent	International Confederation of Midwives
Cherrie Lynn Evans	Meridian Global Health
Ioannis Gallos	UNDP/UNFPA/UNICEF/WHO/World Bank Special Programme of Research, Development and Research Training in Human Reproduction (HRP), Department of Sexual, Reproductive, Maternal, Child, Adolescent Health and Ageing, World Health Organization
David Gomez Canon	Partnership for Maternal, Newborn and Child Health (PMNCH), World Health Organization
Celeste Hibbert	United Nations Population Fund (UNFPA)
Rob Hucker	International Federation of Gynecology and Obstetrics
Anne–Beatrice Kihara	International Federation of Gynecology and Obstetrics
Etienne V Langlois	Partnership for Maternal, Newborn and Child Health (PMNCH), World Health Organization
Frances Longley	International Federation of Gynecology and Obstetrics
Laura Meyer	Reproductive Health Supplies Coalition
Joyce Nganga	WACI Health
Angela Nguku	White Ribbon Alliance, Kenya
Lisa Noguchi	Jhpiego and AlignMNH
Olufemi T Oladapo	UNDP/UNFPA/UNICEF/WHO/World Bank Special Programme of Research, Development and Research Training in Human Reproduction (HRP), Department of Sexual, Reproductive, Maternal, Child, Adolescent Health and Ageing, World Health Organization
Claire Seaward	International Federation of Gynecology and Obstetrics
Jeffrey Michael Smith	Unitaid
Kadidiatou Toure	Partnership for Maternal, Newborn and Child Health (PMNCH), World Health Organization
Andrew Weeks	Department of Women's and Children's Health, University of Liverpool, United Kingdom
Caitlin Williams	UNDP/UNFPA/UNICEF/WHO/World Bank Special Programme of Research, Development and Research Training in Human Reproduction (HRP), Department of Sexual, Reproductive, Maternal, Child, Adolescent Health and Ageing, World Health Organization
